# Protective Effects of Growth Differentiation Factor-6 on the Intervertebral Disc: An In Vitro and In Vivo Study

**DOI:** 10.3390/cells11071174

**Published:** 2022-03-31

**Authors:** Kunihiko Miyazaki, Shingo Miyazaki, Takashi Yurube, Yoshiki Takeoka, Yutaro Kanda, Zhongying Zhang, Yuji Kakiuchi, Ryu Tsujimoto, Hiroki Ohnishi, Tomoya Matsuo, Masao Ryu, Ryosuke Kuroda, Kenichiro Kakutani

**Affiliations:** 1 Department of Orthopaedic Surgery, Kobe University Graduate School of Medicine, Kobe 650-0017, Japan; miya625819@gmail.com (K.M.); yoshiki_tkk@hotmail.com (Y.T.); ykanda221@gmail.com (Y.K.); shouei76@hotmail.com (Z.Z.); yuji_uz_7@yahoo.co.jp (Y.K.); tsujiryu1105@yahoo.co.jp (R.T.); o0717ooo@yahoo.co.jp (H.O.); t.matsuo512@gmail.com (T.M.); smart_thomas0724@yahoo.co.jp (M.R.); kurodar@med.kobe-u.ac.jp (R.K.); kakutani@med.kobe-u.ac.jp (K.K.); 2 Department of Orthopedics Surgery, Anshin Hospital, Kobe 650-0047, Japan

**Keywords:** growth differentiation factor-6 (GDF-6), intervertebral disc (IVD), disc degeneration, annular puncture model, atelocollagen, spine

## Abstract

Growth differentiation factors (GDFs) regulate homeostasis by amplifying extracellular matrix anabolism and inhibiting pro-inflammatory cytokine production in the intervertebral disc (IVD). The aim of this study was to elucidate the effects of GDF-6 on human IVD nucleus pulposus (NP) cells using a three-dimensional culturing system in vitro and on rat tail IVD tissues using a puncture model in vivo. In vitro, Western blotting showed decreased GDF-6 expression with age and degeneration severity in surgically collected human IVD tissues (*n* = 12). Then, in moderately degenerated human IVD NP cells treated with GDF-6 (100 ng/mL), immunofluorescence demonstrated an increased expression of matrix components including aggrecan and type II collagen. Quantitative polymerase chain reaction analysis also presented GDF-6-induced downregulation of pro-inflammatory tumor necrosis factor (TNF)-α (*p* = 0.014) and interleukin (IL)-6 (*p* = 0.016) gene expression stimulated by IL-1β (10 ng/mL). Furthermore, in the mitogen-activated protein kinase pathway, Western blotting displayed GDF-6-induced suppression of p38 phosphorylation (*p* = 0.041) under IL-1β stimulation. In vivo, intradiscal co-administration of GDF-6 and atelocollagen was effective in alleviating rat tail IVD annular puncture-induced radiologic height loss (*p* = 0.005), histomorphological degeneration (*p* < 0.001), matrix metabolism (aggrecan, *p* < 0.001; type II collagen, *p* = 0.001), and pro-inflammatory cytokine production (TNF-α, *p* < 0.001; IL-6, *p* < 0.001). Consequently, GDF-6 could be a therapeutic growth factor for degenerative IVD disease.

## 1. Introduction

Lower back pain is experienced by 85% of adults, notably affecting the global workforce owing to disability [1] which increases the socioeconomic burden [2]. Lower back pain can be caused by multiple factors, including intervertebral disc (IVD) degeneration [3,4]. The IVD comprises a complex discoid structure with the nucleus pulposus (NP) enclosed by the annulus fibrosus (AF) and endplates [4,5,6]. Disc cells have a chondrocytic phenotype to produce extracellular matrix (ECM) components comprising proteoglycans (principally aggrecan) and collagens (primarily type I in the AF and II in the NP) [7].

During IVD degeneration, inflammatory stimuli, primarily involving tumor necrosis factor (TNF)-α, interleukin (IL)-1, and IL-6, modulate the degradation of the ECM through the release of matrix-degrading enzymes, such as matrix metalloproteinase (MMP)-3, MMP-13, and a disintegrin and metalloproteinase with thrombospondin motifs (ADAMTS)-4 [8,9].

Prior studies have demonstrated that growth factors, such as bone morphogenetic protein (BMP)-2, BMP-7, and growth differentiation factor (GDF)-5 (also known as BMP-14), positively affect ECM metabolism in vitro and induce the structural repair of IVD in vivo [10,11,12,13]. Additionally, GDF-6 (also known as BMP-13) plays an essential role in skeletal development by enhancing cartilage growth and suppressing bone formation [14,15,16,17]. In human IVDs, GDF-6 expression is localized into the NP [18]. Further, GDF-6 facilitates the increase in the IVD cell number and water content, thereby contributing to IVD development and homeostasis [16,19,20]. We previously reported that intradiscal GDF-6 injection downregulated the transcript-level expression of pro-inflammatory cytokines— TNF-α, IL-1β, and IL-6—and pain-related molecules—vascular endothelial growth factor, prostaglandin-endoperoxide synthase-2, and nerve growth factor—which are associated with IVD degeneration [21]. Moreover, GDF-6 attenuated pain behavior associated with the pathological status of degenerative IVDs [21]. However, little evidence exists regarding the effects of GDF-6 administration on varying degrees of IVD degeneration, in particular under pro-inflammatory conditions [22,23].

As vehicles for therapeutic drugs, growth factors, and cells, injectable scaffolds using atelocollagen (AC), alginate, hyaluronan, and chitosan have been tested [24]. Clinically, collagen-based scaffolds are often used, as autologous chondrocyte-seeded AC has been implemented for cartilage repair [25]. Type II collagen is the main component of the IVD NP [26]. It serves as not only a structural scaffold for cell proliferation but also as an effective drug delivery carrier for sustained and localized release [26,27]. The AC has been used as a carrier for mesenchymal stem cells, effective in delaying IVD degeneration in vivo [26]. Therefore, in the present study, we hypothesized that the co-administration of GDF-6 with AC would decelerate IVD degeneration. The objective of this study was to elucidate the anabolic and anti-inflammatory effects of GDF-6 on human IVD NP cells with a varying severity of degeneration using an in vitro three-dimensional (3D) culturing system and synergistic effects of GDF-6 and AC for decelerating IVD disruption on rat tail IVD tissues in an in vivo animal model of degeneration induced by annular puncture.

## 2. Materials and Methods

### 2.1. Ethics Statement

All human and animal experiments were performed under the approval and guidance of the Institutional Review Board (160004) and Institutional Animal Care and Use Committee (P200505) at Kobe University Graduate School of Medicine. Written informed consent was obtained from each patient in accordance with the principles of the Declaration of Helsinki and the laws and regulations of Japan.

### 2.2. Human IVD Experiments

#### 2.2.1. Tissues

We carefully identified a piece of human IVD NP tissues from patients who underwent lumbar spine surgery for degenerative disease (*n* = 24: age, 47.1 ± 17.4 (15–70) years; male 12, female 12; Pfirrmann degeneration grade [28], median 3 ± 0.5 (2–4)), to directly obtain protein extracts.

#### 2.2.2. Cells

We immediately isolated human IVD NP cells from surgical waste in patients with degenerative lumbar spine disease. We digested IVD NP tissues in 1% penicillin-streptomycin (26253-84; Nacalai Tesque, Kyoto, Japan)-supplemented Dulbecco’s modified Eagle’s medium (DMEM) (D5796; Sigma-Aldrich, St. Louis, MO, USA) with 10% fetal bovine serum (FBS) (F2442; Sigma-Aldrich) and 0.114% collagenase type II (LS004176; Worthington Biochemical, Lakewood, NJ, USA) for 1 h at 37 °C. We then cultured the separated cells grown up to ~80% confluence as a monolayer in DMEM with 1% penicillin-streptomycin and 10% FBS in an incubator (9000EX; Wakenyaku, Kyoto, Japan) at 37 °C under an atmosphere of 5% CO_2_ and 2% O_2_, to simulate a physiologically hypoxic IVD environment [5]. In this study, we used only first passage cells.

#### 2.2.3. Protein Extraction

To compare protein expression in IVD NP tissues by Western blotting, proteins were extracted from IVD NP tissues. Harvested tissues were homogenized using the MS-100R bead-beating disrupter (Tomy Seiko, Tokyo, Japan) for 30 s twice at 4 °C in the T-PER tissue protein extraction reagent (78510; Thermo Fisher Scientific, Waltham, MA, USA) with protease and phosphatase inhibitors (Protease inhibitor cocktail [25955-11; Nacalai Tesque], Phosphatase inhibitor cocktails 2 [P5726; Sigma-Aldrich], and Phosphatase inhibitor cocktails 3 [P0044; Sigma-Aldrich]). Finally, soluble proteins were collected by centrifugation at 20,000× *g* for 15 min at 4 °C, and the protein concentration was determined using a bicinchoninic acid assay (23227; Thermo Fisher Scientific). Samples were stored at −80 °C.

#### 2.2.4. Western Blotting

To analyze the expression levels of GDF-6 and its receptor, bone morphogenetic protein receptor type II (BMPRII), according to the patient age and Pfirrmann degenerative grade (grades II, *n* = 4; III, *n* = 4; IV, *n* = 4), we used Western blotting. Western blotting was also used to analyze the intracellular expression levels of the IVD NP-associated notochordal markers, brachyury and CD24, and a loading control, actin. Equal amounts of protein (20 μg) were mixed with sample buffer, boiled for 5 min, and loaded onto a 7.5–15% polyacrylamide gel (SDG-581; Bio Craft, Tokyo, Japan). Proteins separated using the Tris (35434-21; Nacalai Tesque)–glycine (12-1210; Sigma-Aldrich)–sodium dodecyl sulfate buffer system were electrotransferred onto a membrane, probed with 1:200−1:1000-diluted primary antibodies (anti-GDF-6 [GTX131252; GeneTex, Irvine, CA, USA], anti-BMPRII [BS-4237R; Bioss, MA], anti-brachyury [sc-17743; Santa Cruz Biotechnology, Santa Cruz, CA, USA], anti-CD24 [sc-11406; Santa Cruz Biotechnology], and anti-actin [A5441; Sigma-Aldrich]) for 12 h at 4 °C, and subsequently with 1:2000-diluted secondary antibodies (anti-rabbit [NA934] and anti-mouse [NA931]; GE Healthcare, Chicago, IL, USA) for 1 h at 25 °C. Protein signals were visualized using enhanced chemiluminescence. Images were obtained using the Chemilumino Analyzer LAS-3000 Mini (Fujifilm, Tokyo, Japan). Band intensity was quantified using ImageJ (https://imagej.nih.gov/ij/, accessed on 19 May 2019).

#### 2.2.5. In Vitro Immunofluorescence

To verify the effects of GDF-6 on human IVD NP cells, we used immunofluorescence to evaluate the expression levels of anabolic molecules. Cells (Pfirrmann degeneration grades III, *n* = 4; IV, *n* = 4) were cultured in a monolayer medium, followed by seeding into a 3D culturing system (Tapered Stencil for Cluster Culture [TASCL] 600 well; Cymss-Bio, Tokyo, Japan) at a density of 1.0 × 10^6^ cells/TASCL to form cell clusters. After 48 h, cell clusters were treated with GDF-6 (100 ng/mL, group G) (AF-120-04; PeproTech, Rocky Hill, NJ), IL-1β (10 ng/mL, group I) (ALX-520-001; Enzo Life Science, Farmingdale, NY, USA), or both treatments (group G + I). The IL-1β is a pro-inflammatory cytokine closely related to the pathogenesis and severity of IVD degeneration [29]. The concentrations of GDF-6 and IL-1β were adjusted according to protocols described previously [30,31,32,33].

**Figure 1:**
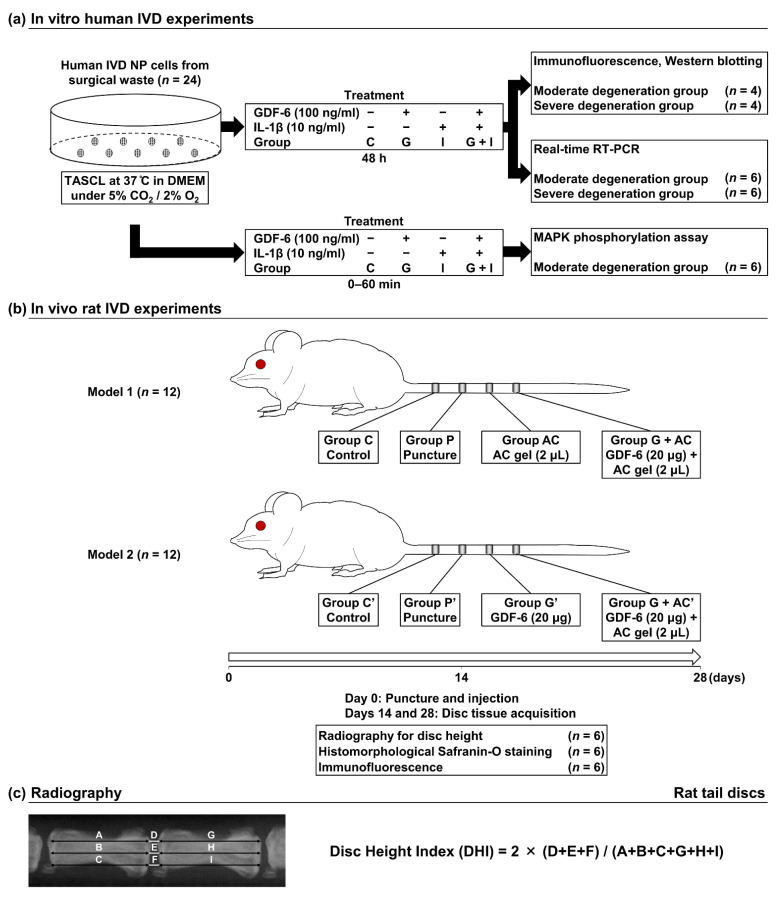
(**a**) Schematic illustration of in vitro experimental regimens. First passage, ~80%-confluent human intervertebral disc (IVD) nucleus pulposus (NP) cells from surgical waste in patients with degenerative lumbar spine disease (total *n* = 24) were cultured as a monolayer in Dulbecco’s modified Eagle’s medium (DMEM) with 1% penicillin-streptomycin (PS) and 10% fetal bovine serum (FBS) at 37 °C under 5% CO_2_ and 2% O_2_, followed by seeding into a three-dimensional culturing system (Tapered Stencil for Cluster Culture [TASCL]) to form cell clusters. Cell clusters were treated with growth differentiation factor (GDF)-6 (100 ng/mL, group G), interleukin (IL)-1β (10 ng/mL, group I), or both treatments (group G + I). Non-treated cell clusters were used as the control (group C). Then, cell clusters were analyzed by immunofluorescence, Western blotting (Pfirrmann degeneration grades III [moderate], *n* = 4; IV [severe], *n* = 4), real-time reverse transcription–polymerase chain reaction (RT–PCR) (Pfirrmann degeneration grades II–III [moderate], *n* = 6; IV [severe], *n* = 6), or Western blotting for the phosphorylation of p38 mitogen-activated protein kinase (MAPK) pathways (Pfirrmann degeneration grades II–III [moderate], *n* = 6). (**b**) Schematic illustration of in vivo experimental regimens. In 12-week-old male Sprague–Dawley rat tails (*n* = 24), annular punctures at C8–C9, C9–C10, and C10–C11 discs were performed, while the C7–C8 disc was later collected as the non-treated control (groups C and C′). Immediately following the puncture, phosphate-buffered saline (PBS) vehicle (2 μL per disc, group P), atelocollagen (AC) (2 μL per disc, group A), or GDF-6 + AC (20 μg in 2-μL AC gel per disc, group G + AC) was randomly injected into the center of the IVD NP (Model 1, *n* = 12). In the other experiment, PBS vehicle (2 μL per disc, group P′), GDF-6 (20 μg in 2-μL PBS per disc, group G′), or GDF-6 + AC (20 μg in 2-μL AC per disc, group G + AC′) was randomly injected similarly (Model 2, *n* = 12). Disc height evaluation, histomorphology, and immunofluorescence were performed at 14 and 28 days following the puncture (*n* = 6/time point). (**c**) Measurements of the disc height index (DHI). The DHI was calculated by measuring, averaging, and normalizing the disc height to the adjacent vertebral body heights at the anterior, middle, and posterior portions.

Cell clusters, formed with TASCL 48 h after the administration, were incubated with 1:200-diluted primary antibodies against IVD NP-associated CD24, aggrecan (ab3773; Abcam, Cambridge, UK), and type II collagen (sc-7764; Santa Cruz Biotechnology) overnight at 4 °C. Cell clusters were further treated with Alexa Fluor 488-conjugated (A21200; Thermo Fisher Scientific), Alexa Fluor 568-conjugated (A21468; Thermo Fisher Scientific), and Alexa Fluor 647-conjugated (A31573; Thermo Fisher Scientific) secondary antibodies for 1 h at 25 °C. Finally, cells were counterstained with 4′,6-diamidino-2-phenylindole (DAPI: D1306; Thermo Fisher Scientific). Immunofluorescence images of cell clusters on TASCL were obtained using a microscope (BZ-X700; Keyence, Osaka, Japan). The relative fluorescence intensity of 10 randomly selected cell clusters per image was measured using a microscope and BZ-X700 software according to a prior study [34]. Portions of cell clusters were additionally analyzed by Western blotting (Figure 1a).

#### 2.2.6. RNA Isolation and Real-Time Reverse Transcription–Polymerase Chain Reaction

To verify the effects of GDF-6 on human IVD NP cells, we used real-time reverse transcription–polymerase chain reaction (RT–PCR) for the messenger RNA (mRNA) expression levels of anabolic factors and pro-inflammatory cytokines. Initially, cells (Pfirrmann degeneration grades II, *n* = 2; III, *n* = 4; IV, *n* = 6) were divided into four groups, cultured in TASCL microplates, and treated with GDF-6 and IL-1β (control, group C; 100-ng/mL GDF-6, group G; 10-ng/mL IL-1β, group I; 100-ng/mL GDF-6 + 10-ng/mL IL-1β, group G + I). Total RNA was extracted using the RNeasy mini kit (74104; Qiagen, Hilden, Germany), and RNA (0.2 μg) was reverse transcribed with High Capacity cDNA Reverse Transcription Kit (4368814; Thermo Fisher Scientific). The mRNA expression of anabolic factors (ACAN encoding aggrecan and COL2A1 encoding type II collagen) and pro-inflammatory cytokines (TNFA encoding TNF-α, IL-6, MMP-3, and ADAMTS-4) relative to glyceraldehyde 3-phosphate dehydrogenase (GAPDH) was assessed through RT–PCR using SYBR Green (4367659; Thermo Fisher Scientific). The primer sequences are listed in Table 1, obtained from published reports [35,36] and purchased from Takara Bio (Shiga, Japan) and Thermo Fisher Scientific. Measurements were performed using the ABI Prism 7500 RT–PCR system (Thermo Fisher Scientific). Melt curve analysis was performed using the Dissociation Curves software to ensure that only a single product was amplified. The relative mRNA expression was analyzed using the 2^−ΔΔCt^ method, and the vehicle control value was set at 1.0. In quantitative RT–PCR analysis, the effects of treatment on target gene expression were calculated as relative values of the vehicle control. Sample analysis for each patient was performed in duplicate, and single data values were obtained by averaging the values of these replicates. Experiments were conducted six times using six patient samples (*n* = 12) (Figure 1a).

#### 2.2.7. p38 Mitogen-Activated Protein Kinase Phosphorylation Assay

Activation of the p38 mitogen-activated protein kinase (MAPK) pathway owing to increased IL-1β levels during the progression of IVD degeneration is an early event [31]. Hence, we investigated the phosphorylation of MAPK pathways by GDF-6 and pro-inflammatory IL-1β stimulation in cultured moderately degenerated human IVD NP cells (Pfirrmann degeneration grades II, *n* = 2; III, *n* = 4). Initially, we selected five time points (0, 15, 30, 45, and 60 min) to determine the time point at which the activation of the MAPK signaling pathway peaked following the stimulation of cultured cells with 10-ng/mL IL-1β using Western blotting. Furthermore, we co-treated the cultured cells with 100-ng/mL GDF-6 and 10-ng/mL IL-1β and evaluated the effects of GDF-6 on the p38 MAPK signaling pathway at the peak time point. In addition, Western blotting was performed six times using six patient samples to evaluate the phosphorylation of p38 MAPK (#9926; MAPK Family Antibody Sample Kit, Cell Signaling, Danvers, MA, USA). Representative immunoblots of six similar results are presented (*n* = 6) (Figure 1a).

### 2.3. Rat IVD Experiments

#### 2.3.1. Animals

Twelve-week-old male Sprague–Dawley rat tails were used to develop a model of IVD degeneration (*n* = 24: weight, 412.2 ± 15.4 (380–444) g) [37]. Under general anesthesia, we used a 20-gauge (G) needle to puncture the skin of rat caudal vertebra to the center of the NP at a depth of 5 mm. The needle was rotated 360° and held in that position for 30 s before removal. Moreover, we adjusted the AC to be injected into the IVD and prepared 8 mL of cooled atelocollagen (621092; Koken, Tokyo, Japan) solution composed of 0.3% type II collagen, as described in a published report [26], by adding 1 mL of low-concentration glucose DMEM, 0.1 mL of 4-(2-hydroxyethyl)-1-piperazineethanesulfonic acid (H0887; Sigma-Aldrich), and 0.1 mL of 2.2% NaHCO_3_ (28-1850-5; Sigma-Aldrich). Punctures at C8–C9, C9–C10, and C10–C11 were performed to assess effects of GDF-6 and AC [26].

Immediately following the initial puncture, either phosphate-buffered saline (PBS) vehicle (2 μL per disc, group P), AC (2 μL per disc, group A), or GDF-6 (20 μg in 2-μL AC gel per disc, group G + AC) was randomly injected into 12 rats at the center of the IVD NP, using a 27-G needle (MS-N05; Ito Corporation, Shizuoka, Japan). The amount of GDF-6 administered per rat was set to 20 μg using the body weight ratio from a prior study on rabbits (100 μg/3.3–4.5-kg rabbit) [21]. Disc height evaluation, histomorphology, and immunofluorescence were performed at 14 and 28 days following the puncture (model 1, *n* = 12).

To confirm the effects of AC co-administration, the remaining rats were similarly injected with either PBS vehicle (2 μL per disc, group P′), GDF-6 (20 μg in 2-μL PBS per disc, group G′), or GDF-6 (20 μg in 2-μL AC per disc, group G + AC′) following the initial puncture. Subsequently, disc height evaluation, histomorphology, and immunofluorescence were performed similarly (model 2, *n* = 12) (Figure 1b).

#### 2.3.2. Radiography

Lateral radiographs were obtained using the VPX-30E (Toshiba Medical Supply, Tokyo, Japan) system and IXFR film (Fujifilm) (exposure time, 40 s; distance, 40 cm; current, 3 mA; voltage, 35 kV). Disc height was measured using ImageJ, normalized to adjacent vertebral body heights as the disc height index (DHI), presented as the percentage of preoperative DHI (%DHI = [postoperative DHI/preoperative DHI] × 100), as described previously [38], and normalized to the intact IVD as normalized %DHI (normalized %DHI = [experimental %DHI/intact %DHI] × 100, *n* = 6 per group) (Figure 1c).

#### 2.3.3. Paraffin-Embedded Tissue Preparation

After euthanasia, the rat caudal functional spinal units of the vertebral body–IVD–vertebral body was obtained, fixed in 4% paraformaldehyde (163-20145; Wako Osaka, Japan) for 1 day, demineralized in 10% ethylenediaminetetraacetic acid (345-01865; Wako) for 7 days, embedded in paraffin, and cut to obtain a mid-sagittal 7-μm section for histomorphology and immunofluorescence.

#### 2.3.4. Histomorphology

Safranin-O (S-0145; Tokyo Chemical Industry, Tokyo, Japan), fast green (10720; Chroma Gesellschaft Schmidt, Munster, Germany), and hematoxylin (30002; Muto Tokyo, Japan) staining were used to visualize the distribution of proteoglycans. Images of the sections were captured with the BZ-X700 microscope and graded by three individual examiners from 0 (non-degenerative) to 16 (severely degenerative) for NP morphology, NP cellularity, NP-AF boundary, AF morphology, and endplate, according to a reported histological degeneration scale (*n* = 6 per group) [39].

#### 2.3.5. In Vivo Immunofluorescence

Immunofluorescence was performed to assess ECM component and pro-inflammatory cytokine production. After antigen-retrieval, permeabilization, and blocking, multi-color immunofluorescence was produced with 1:200-diluted primary antibodies against an IVD NP-associated notochordal marker (CD24), ECM components (aggrecan and type II collagen), and pro-inflammatory cytokines (TNF-α [NBP2-34372; Novusbio, Centennial, CO, USA] and IL-6 [AF506; R & D Systems, Minneapolis, MN, USA]) for 12 h at 4 °C, followed by incubation with 1:400-diluted Alexa Fluor 488 (A-21200; Thermo Fisher Scientific), 594 (A-21468; Thermo Fisher Scientific), and 647 (A-31573; Thermo Fisher Scientific) secondary antibodies for 1 h at 25 °C. The DAPI was used for counterstaining. Images were captured by fluorescence spectroscopy using the BZ-X700 microscope. The number of positive cells was counted in four random high-power field (×400) using the BZ-X700 software. Immunopositivity was calculated as a percentage relative to DAPI-positive cells (*n* = 6 per group).

### 2.4. Statistical Analysis

Statistical analysis was performed using IBM SPSS Statistics 23.0 software (IBM Corp., Armonk, NY, USA). Data were expressed as the mean ± standard deviation, except for histological grading scores, which were reported as the median with the range. To assess the effects of changes in culture durations and experimental conditions, one-way analysis of variance (ANOVA) with the Tukey–Kramer post-hoc test was performed with a significance level of *p* < 0.050 with the normality assumption.

## 3. Results

### 3.1. In Vivo Human IVD Experiments

**Figure 2:**
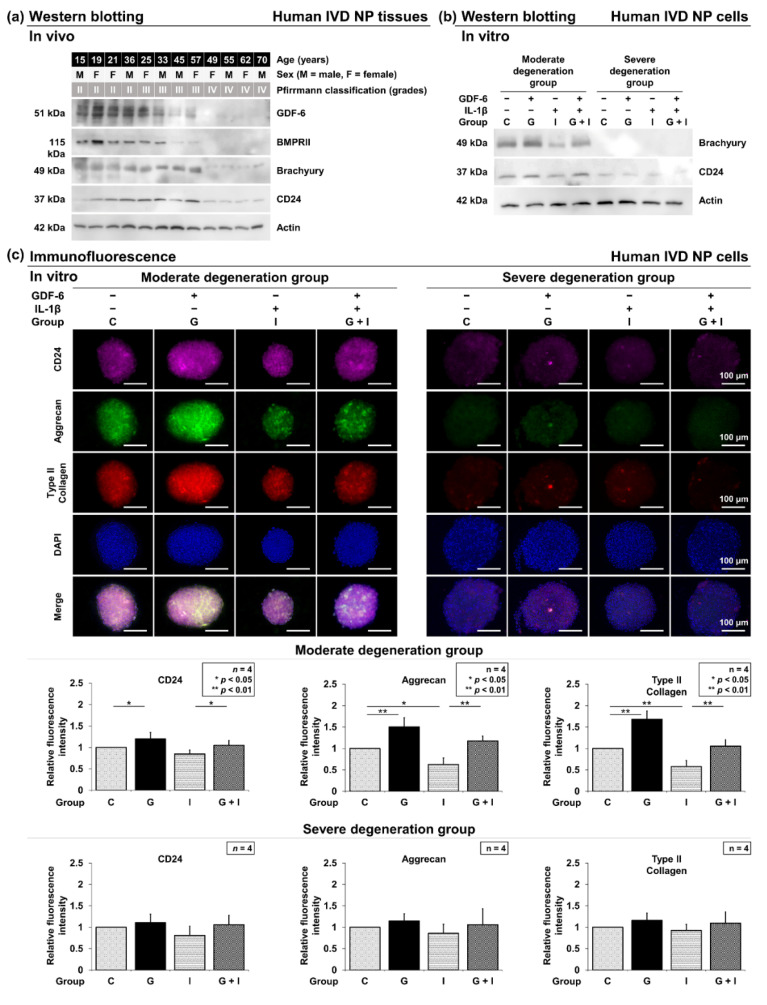
(**a**) Variation in growth differentiation factor-6 (GDF-6) levels with aging and intervertebral disc (IVD) degeneration. Levels of GDF-6, bone morphogenetic protein receptor type II (BMPRII), IVD nucleus pulposus (NP)-associated notochordal brachyury, CD24, and actin (used as a loading control) were examined by Western blotting in protein extracts from surgically obtained human IVD NP tissues. Samples were randomly selected (*n* = 12). (**b**) Validation of the IVD NP phenotype in cell clusters. Levels of IVD NP-associated notochordal brachyury and CD24 were evaluated by Western blotting in protein extracts from human IVD NP cells. Immunoblots shown are representative of in vitro human IVD experiments, as described below, with similar results. (**c**) Effects of growth differentiation factor-6 (GDF-6) treatment on CD24, aggrecan, and type II collagen expression in human IVD NP cells (Pfirrmann degeneration grades III and IV, *n* = 4). Cells were cultured in a monolayer medium, followed by seeding in a three-dimensional culturing system (Tapered Stencil for Cluster Culture [TASCL]) at 1.0 × 10^6^ cells/TASCL to form cell clusters. After 48-h culture, cell clusters were treated with solvent as a control (group C, set as 1.0), GDF-6 treatment (100 ng/mL, group G), pro-inflammatory interleukin (IL)-1β (10 ng/mL, group I), or both GDF-6 and IL-1β (group G + I). Then, CD24 (purple), aggrecan (green), type II collagen (red), nuclear 4′,6-diamidino-2-phenylindole (DAPI) (blue), and merged signals of cell clusters on TASCL were obtained using a fluorescence microscope. Relative fluorescence intensity of 10 randomly selected cell clusters per image was measured. Data are the mean ± standard deviation. (*n* = 4). One-way analysis of variance with the Tukey–Kramer post-hoc test was used. Significant differences were set as * *p* < 0.050 and ** *p* < 0.010.

Western blotting was used to evaluate GDF-6 expression in surgically collected human IVD NP tissues based on the patient’s age and Pfirrmann degeneration grade (*n* = 12). The expression of GDF-6 tended to decrease with increasing age and degeneration grade. Additionally, BMPRII and notochordal brachyury and CD24 diminished in protein expression with aging and degeneration. Levels of GDF-6 were lower in Pfirrmann grade-IV IVDs than in grade-II and grade-III tissues. These results indicate that the roles of GDF-6 could differ depending on the degeneration grade. Therefore, we divided these human tissue samples into two experimental groups: grades II and III as the moderate degeneration group and grade IV as the severe degeneration group (Figure 2a).

### 3.2. In Vitro Human IVD Experiments

#### 3.2.1. Effects of GDF-6 Treatment on CD24, Aggrecan and Type II Collagen

Following Western blotting in human IVD NP tissues, multi-color immunofluorescence was performed in both moderate and severe degeneration groups to confirm the levels of IVD NP-associated notochordal CD24 and anabolic aggrecan and type II collagen in GDF-6-treated IVD NP cell clusters under pro-inflammatory IL-1β stimulation. The phenotype of human IVD NP cells used in all in vitro experiments of this study was confirmed by Western blotting for cell markers (Brachyury and CD24) (Figure 2b). In the moderate degeneration group, immunofluorescence identified significantly higher immunoreactivity for CD24 (*p* = 0.028), aggrecan (*p =* 0.001), and type II collagen (*p* < 0.001) in group G than in group C. Although IL-1β supplementation resulted in significantly lower expression of CD24 (*p* = 0.029), aggrecan (*p* = 0.013) and type II collagen (*p* = 0.006) in group I, IL-1β-induced suppression of CD24 (*p* = 0.030), aggrecan (*p* = 0.001), and type II collagen (*p* = 0.002) levels was significantly rescued by the supplementation of GDF-6 in group G + I. However, no significant difference was observed between groups G, I, and C in the severe degeneration group (Figure 2c).

#### 3.2.2. Effects of GDF-6 Treatment on TNF-α, IL-6, MMP-3, and ADAMTS-4 Gene Expression

**Figure 3:**
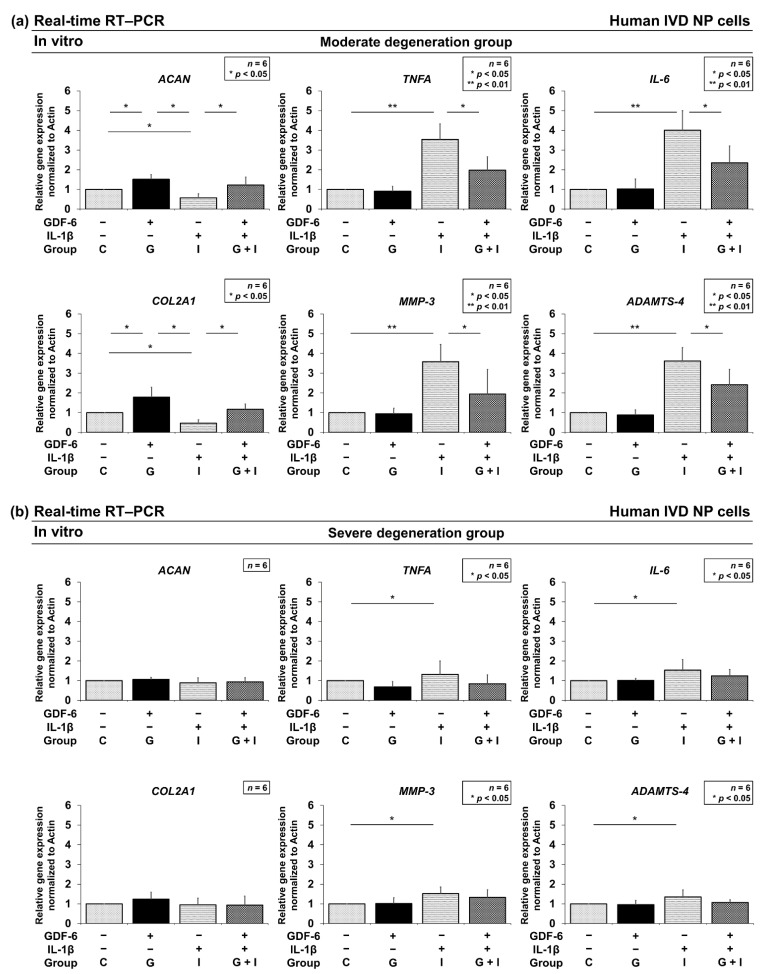
Effects of growth differentiation factor-6 (GDF-6) treatment on anabolic *ACAN* encoding aggrecan, *COL2A1* encoding type II collagen, pro-inflammatory *TNFA* encoding tumor necrosis factor-α (TNF-α), *interleukin* (*IL*)*-6*, catabolic *matrix metalloproteinases-3* (*MMP-3*), and *a disintegrin and metalloproteinase with thrombospondin motifs-4* (*ADAMTS-4*) gene expression in intervertebral disc nucleus pulposus cells. Cells of the moderate degeneration group (**a**) and severe degeneration group (**b**) were seeded on Tapered Stencil for Cluster Cultures and treated with solvent as a control (group C; set as 1.0), 100-μg/mL GDF-6 (group G), 10-ng/mL pro-inflammatory IL-1β (group I), or both (group G + I) for 48 h. Gene expression of *ACAN*, *COL2A1*, *TNF*A, *IL*-6, *MMP*-3, and *ADAMTS-4* relative to *glyceraldehyde 3-phosphate dehydrogenase* (*GAPDH*) was assessed through real-time reverse transcription–polymerase chain reaction using SYBR Green. Data are the mean ± standard deviation as the fold change relative to the control (group C) (*n* = 6). One-way repeated-measures analysis of variance with the Tukey–Kramer post-hoc test was used. Significant differences were set as * *p* < 0.050 and ** *p* < 0.010.

To analyze the mRNA expression levels of anabolic factors and pro-inflammatory cytokines, real-time RT–PCR was performed. Quantitative RT–PCR identified significantly higher gene expression of *ACAN* encoding aggrecan (*p* = 0.031) and *COL2A1* encoding type II collagen (*p* = 0.024) in group G than in group C with the moderate degeneration group (Figure 3a). However, no significant difference was observed between groups G and C with the severe degeneration group (Figure 3b). Furthermore, significantly lower gene expression of *TNFA* encoding TNF-α (*p* = 0.014), *IL-6* (*p =* 0.016), *MMP-3* (*p* = 0.038), and *ADAMTS-4* (*p* = 0.017) was found in group G + I than in group I with the moderate degeneration group. No significant difference was observed between groups I and G + I with the severe degeneration group. These results suggest that GDF-6 has anti-inflammatory effects under pro-inflammatory conditions. Thus, we further investigated the involvement of the MAPK pathway.

#### 3.2.3. Effects of GDF-6 on p38 Phosphorylation under Pro-Inflammatory Conditions

**Figure 4:**
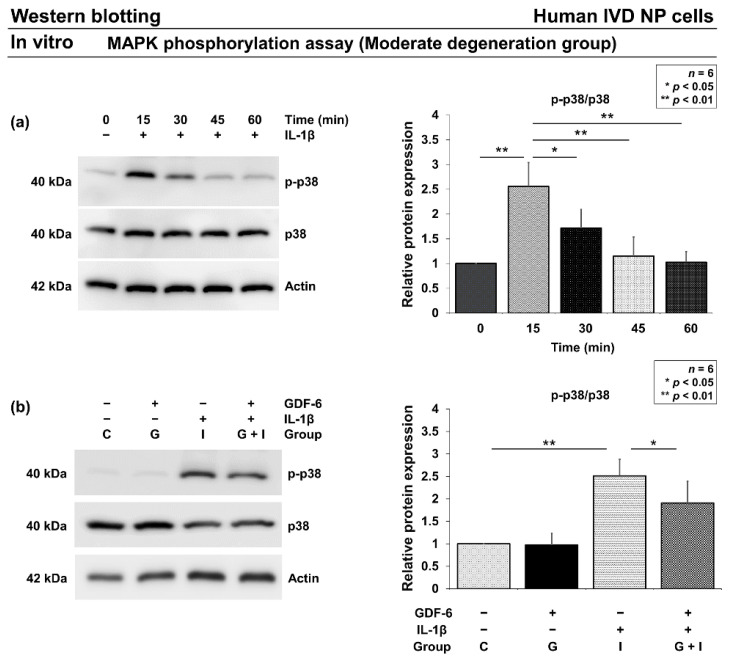
Effects of growth differentiation factor-6 (GDF-6) on p38 phosphorylation under pro-inflammatory condition. (**a**) Time-course change in p-p38 protein levels relative to p38. We stimulated human intervertebral disc (IVD) nucleus pulposus (NP) cells with 10-ng/mL interleukin (IL)-1β and measured the protein levels of p-p38 and p38 at different time points, i.e., 0 (set as 1.0), 15, 30, 45, and 60 min. (**b**) p-p38 protein levels relative to p38 in IVD NP cells treated with phosphate-buffered saline (group C; set as 1.0), GDF-6 (100 µg/mL, group G), IL-1β (10 ng/mL, group I), and both (group G + I) for 15 min. Data are the mean ± standard deviation. Results from six independent experiments were analyzed using one-way repeated-measures analysis of variance with the Tukey–Kramer post-hoc test (*n* = 6). Significant differences are set as * *p* < 0.050 and ** *p* < 0.010.

Based on real-time RT–PCR results, we further investigated the involvement of GDF-6 in the MAPK pathway. In human IVD NP cells of the moderate degeneration group, Western blotting found increased p38 phosphorylation with a peak at 15 min after IL-1β supplementation (Figure 4a). Then, after the 15-min stimulation, Western blotting further identified a significant partial reduction in elevated p38 phosphorylation in group G + I compared with group I (*p* = 0.041) (Figure 4b).

### 3.3. In Vivo Rat IVD Experiments

#### 3.3.1. Effect of Co-Administration of GDF-6 and AC on Radiographic Disc Height in a Rat Tail IVD Annular Puncture Model

**Figure 5:**
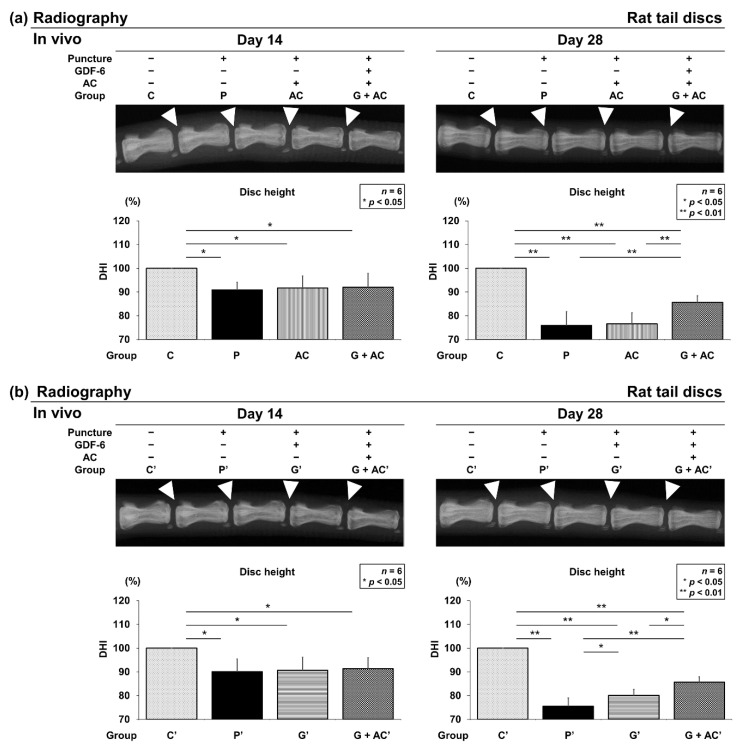
Effects of co-administration of growth differentiation factor-6 (GDF-6) and atelocollagen (AC) on radiographic disc height in a rat tail intervertebral disc annular puncture model. A 20-G needle was used to puncture the rat caudal disc at a depth of 5 mm from the skin to the center of nucleus pulposus. The needle was rotated 360° and held in that position for 30 s before extraction to establish an intervertebral disc degeneration model. Following the initial puncture, (**a**) either vehicle (phosphate-buffered saline [PBS]; 2 μL per disc, group P), atelocollagen (AC; 2-μL AC per disc, group AC), or GDF-6 (20 μg in 2-μL AC gel per disc, group G + AC) or (**b**) vehicle (PBS; 2 μL per disc, group P′), GDF-6 (20 μg in 2-μL PBS per disc, group G′), or GDF-6 (20 μg in 2-μL AC gel per disc, group G + AC′) was randomly injected. Lateral radiographs of the rat tail were obtained on days 14 and 28 postoperatively, and the disc height index (DHI) was calculated by measuring, averaging, and normalizing the disc height compared to the adjacent vertebral body heights in the anterior, middle, and posterior regions. We designated the control without puncture as 100% (groups C and C′), and compared the four groups (C, P, A, G + AC) and (C′, P′, G′, G + AC′). Data are the mean ± standard deviation (*n* = 6). One-way analysis of variance with the Tukey–Kramer post-hoc test was used. Significant differences are set as * *p* < 0.050 and ** *p* < 0.010.

Following the identification of anti-inflammatory characteristics of human IVD NP cells under GDF-6 treatment, we designed an in vivo study using a well-established rat tail IVD annular puncture model characterized by its high accessibility and reproducibility. Group P showed a significantly lower DHI on days 14 (90.9 ± 3.2%, *p* = 0.011) and 28 (75.9 ± 5.9%, *p* < 0.001) after surgery than group C. Moreover, group AC exhibited a significantly lower DHI on days 14 (91.7 ± 5.0%, *p* = 0.022) and 28 (76.6 ± 4.6%, *p* < 0.001) than group C. Similarly, group G + AC also had significantly lower DHI on days 14 (92.0 ± 5.9%, *p* = 0.027) and 28 (85.7 ± 2.8%, *p* < 0.001) than group C. However, DHI at postoperative 28 days in group G + AC was significantly higher than that in groups P (*p* = 0.005) and AC (*p* = 0.009) (Figure 5a). Groups P′, G′, and G + AC′ presented significantly lower DHI on days 14 and 28 than group C′. At postoperative 28 days, DHI in group G + AC′ was significantly higher than in groups P′ (*p* < 0.001) and G′ (*p* = 0.012). In addition, DHI in group G′ was significantly higher compared with that in group P′ (*p* = 0.037) (Figure 5b).

#### 3.3.2. Effect of Co-Administration of GDF-6 and AC on Histological IVD Degeneration in a Rat Tail Puncture Model

Safranin-O, fast green, and hematoxylin staining were performed to assess the general IVD morphology with proteoglycan distribution. Control IVDs had round NPs at 14 and 28 days after the start of the experiment, with a clear boundary observed between the AF and NP.

**Figure 6:**
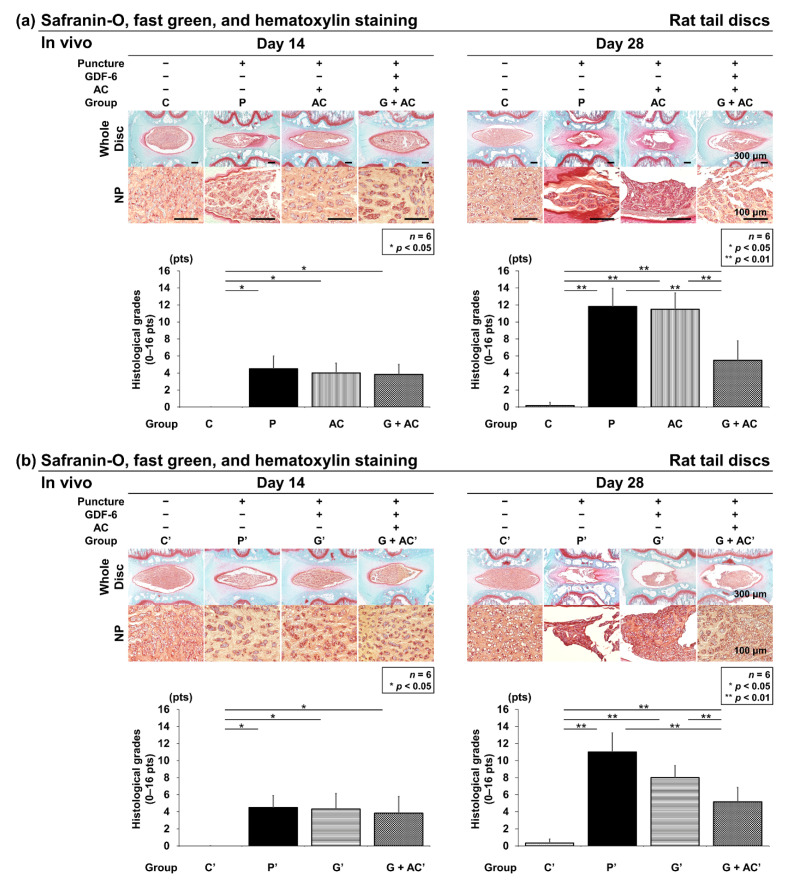
Effects of co-administration of growth differentiation factor-6 (GDF-6) and atelocollagen (AC) on histological intervertebral disc (IVD) degeneration in a rat tail puncture model. After establishment of the IVD degeneration model, (**a**) either vehicle (phosphate-buffered saline [PBS]; 2 μL per disc, group P), AC (2-μL AC per disc, group AC), or GDF-6 (20 μg in 2-μL AC gel per disc, group G + AC) or (**b**) vehicle (PBS; 2 μL per disc, group P′), GDF-6 (20 μg in 2-μL PBS per disc, group G′), or GDF-6 (20 μg in 2-μL AC gel per disc, group G + AC′) was randomly injected. The IVD degeneration was assessed by Safranin-O and fast green staining of the IVDs at 14 and 28 days after injection in low-power fields (×100). We designated the control without puncture (groups C and C′), and compared the four groups (C, P, A, G + AC) and (C′, P′, G′, G + AC′). Data are the mean ± standard deviation (*n* = 6). One-way analysis of variance with the Tukey–Kramer post-hoc test was used. Significant differences are set as * *p* < 0.050 and ** *p* < 0.010.

Nucleated cells were evenly distributed in the NP area with ECM proteoglycans organized into thin striations. In the puncture groups (P, AC, and G + AC), AF tissues presented a ruptured or tortuous pattern, a slight decrease in cell number, and slight condensation of ECM at 14 days after puncture. Moreover, histologically significant degeneration was observed in the puncture groups (P, AC, and G + AC) compared to the control group (group C) (C: 0.0 ± 0.0; P: 4.5 ± 1.5, *p* < 0.001; AC: 4.0 ± 1.2, *p* < 0.001; G + AC: 3.8 ± 1.2, *p* < 0.001) (Figure 6a). In the puncture groups (P′, G′, and G + AC′), histologically significant degeneration was observed compared to the control group (group C′) (C′: 0.0 ± 0.0; P′: 4.5 ± 1.4, *p* = 0.001; G′: 4.3 ± 1.8, *p* = 0.001; G + AC′: 3.8 ± 2.0, *p* = 0.003) (Figure 6b). However, no significant difference was observed between the puncture groups.

Then, 28 days after puncture, degeneration was significantly more progressive in the puncture groups (P, AC, and G + AC) than in group C (C: 0.2 ± 0.4; P: 11.8 ± 2.1, *p* < 0.001; AC: 11.5 ± 1.9, *p* < 0.001; G + AC: 5.5 ± 2.3, *p* < 0.001) (Figure 6a). Similarly, in the puncture groups (P′, G′, and G + AC′), degeneration was significantly more severe than in group C′ (C′: 0.3 ± 0.5; P′: 11.0 ± 2.2, *p* < 0.001; G′: 8.0 ± 1.4, *p* < 0.001; G + AC′: 5.2 ± 1.7, *p* < 0.001) (Figure 6b). Although each group exhibited progressive IVD degeneration 28 days after puncture compared with 14 days after puncture, the histological grade in group G + AC was significantly lower than in groups P (*p* < 0.001) and AC (*p* < 0.001) (Figure 6a). Furthermore, group G′ presented a significantly lower degeneration grade than group P′ (*p* = 0.029); group G + AC′ also presented a significantly lower grade than groups P′ (*p <* 0.001) and G′ (*p =* 0.041) (Figure 6b).

#### 3.3.3. Effects of Co-Administration of GDF-6 and AC on Aggrecan and Type II Collagen Expression in a Rat Tail Puncture Model

**Figure 7:**
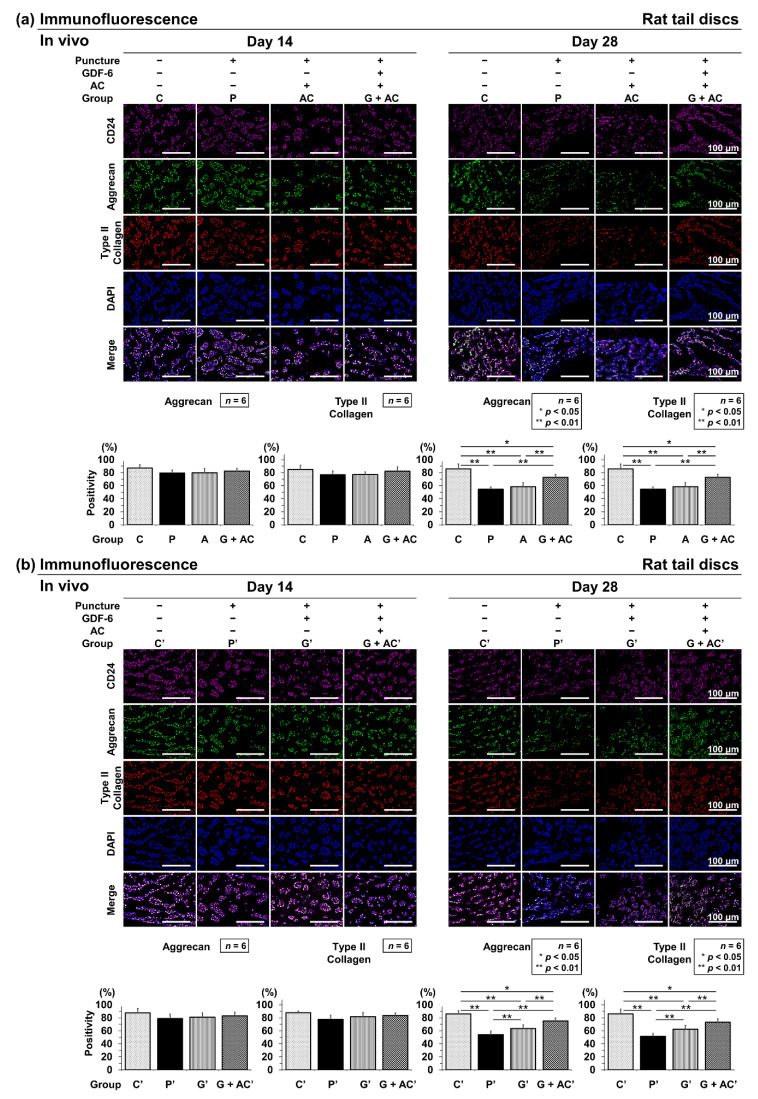
(**a**) Effects of co-administration of growth differentiation factor-6 (GDF-6) and atelocollagen (AC) on aggrecan and type II collagen expression compared with AC alone in a rat tail puncture model. After establishment of the intervertebral disc degeneration model, vehicle (phosphate-buffered saline [PBS]; 2 μL per disc, group P), AC (2-μL AC per disc, group AC), or GDF-6 (20 μg in 2-μL AC gel per disc, group G + AC) was randomly injected. Extracellular matrix components were assessed using immunofluorescence (aggrecan: green, type II collagen: red, CD24: purple, 4′,6-diamidino-2-phenylindole [DAPI]: blue, and merged signals) of the discs at 14- and 28-days post-injection in high-power fields (×400). Immunopositivity was calculated as a percentage relative to DAPI-positive cells. We designated the control without puncture (groups C and C′), and compared the four groups (C, P, A, G + AC) and (C′, P′, G′, G + AC′). Data are expressed as the mean ± standard deviation (*n* = 6). One-way analysis of variance (ANOVA) with the Tukey–Kramer post-hoc test was used. Significant differences are set as * *p* < 0.050 and ** *p* < 0.010. (**b**) Effect of co-administration of GDF-6 and AC on aggrecan and type II collagen expression compared with GDF-6 alone in a rat tail puncture model. After establishment of the intervertebral disc (IVD) degeneration model, vehicle (PBS; 2 μL per disc, group P′), GDF-6 (20 μg in 2-μL PBS per disc, group G′), or GDF-6 (20 μg in 2-μL AC gel per disc, group G + AC′) was randomly injected. Extracellular matrix components were assessed using immunofluorescence (aggrecan: green, type II collagen: red, CD24: purple, 4′,6-diamidino-2-phenylindole [DAPI]: blue, and merged signals) of the IVDs at 14 and 28 days after injection in high-power fields (×400). Immunopositivity was calculated as a percentage relative to DAPI-positive cells. We designated the control without puncture (groups C and C′), and compared the four groups (C, P, A, G + AC) and (C′, P′, G′, G + AC′). Data are the mean ± standard deviation (*n* = 6). One-way ANOVA with the Tukey–Kramer post-hoc test was used. Significant differences are set as * *p* < 0.050 and ** *p* < 0.010.

Immunopositivity was calculated as a percentage relative to DAPI-positive cells. In the control, aggrecan-positive cells were widely observed at both time points (C: 14 days, 87.1 ± 5.1%; 28 days, 85.9 ± 7.4%) and C′: 14 days, 87.6 ± 6.4%; 28 days, 86.0 ± 4.6%) (Figure 7a). Type II collagen-positive cells were also abundant at both time points (C: 14 days, 85.0 ± 6.3%; 28 days, 84.5 ± 6.3%) and C′: 14 days, 87.9 ± 2.4%; 28 days, 86.1 ± 7.1%) (Figure 7b). Therefore, approximately 85–90% of IVD NP cells had abundant ECM components.

Although a slight decrease in aggrecan-positive cell percentage was observed at 14 days after puncture in the puncture groups (P, AC, and G + AC), no significant difference was observed compared with group C (P: 79.5 ± 4.9%, AC: 79.8 ± 6.8%, and G + AC: 82.5 ± 4.2%) (Figure 7a). Similarly, in the puncture groups (P′, G′, and G + AC′), a decrease in the immunopositivity of aggrecan (P′: 78.9 ± 6.6%, G′: 81.2 ± 6.6%, G + AC′: 83.1 ± 5.7%) was observed compared with group C′, although it did not reach statistical significance (Figure 7a,b). Type II collagen-positive cell percentage presented similar results (P: 76.9 ± 5.8%, AC: 77.1 ± 4.2%, and G + AC: 82.2 ± 6.8% and P′: 77.7 ± 6.2%, G′: 81.3 ± 5.9%, and G + AC′: 83.5 ± 3.7%) (Figure 7a,b).

Then, 28 days after puncture, the decrease in group G + AC compared with in groups P (aggrecan; *p* < 0.001, type II collagen; *p* = 0.001) and AC (aggrecan; *p* = 0.004, type II collagen; *p* = 0.003) (Figure 7a) and that in group G + AC′ compared with in groups P′ (aggrecan; *p* < 0.001, type II collagen; *p* < 0.001) and G′ (aggrecan; *p* = 0.001, type II collagen; *p* = 0.045) were significantly suppressed. The decrease in group G′ compared with in group P′ (aggrecan; *p* = 0.031, type II collagen; *p* = 0.029) was also attenuated (Figure 7b).

#### 3.3.4. Effects of Co-Administration of GDF-6 and AC on TNF-α and IL-6 Expression in a Rat Tail Puncture Model

**Figure 8:**
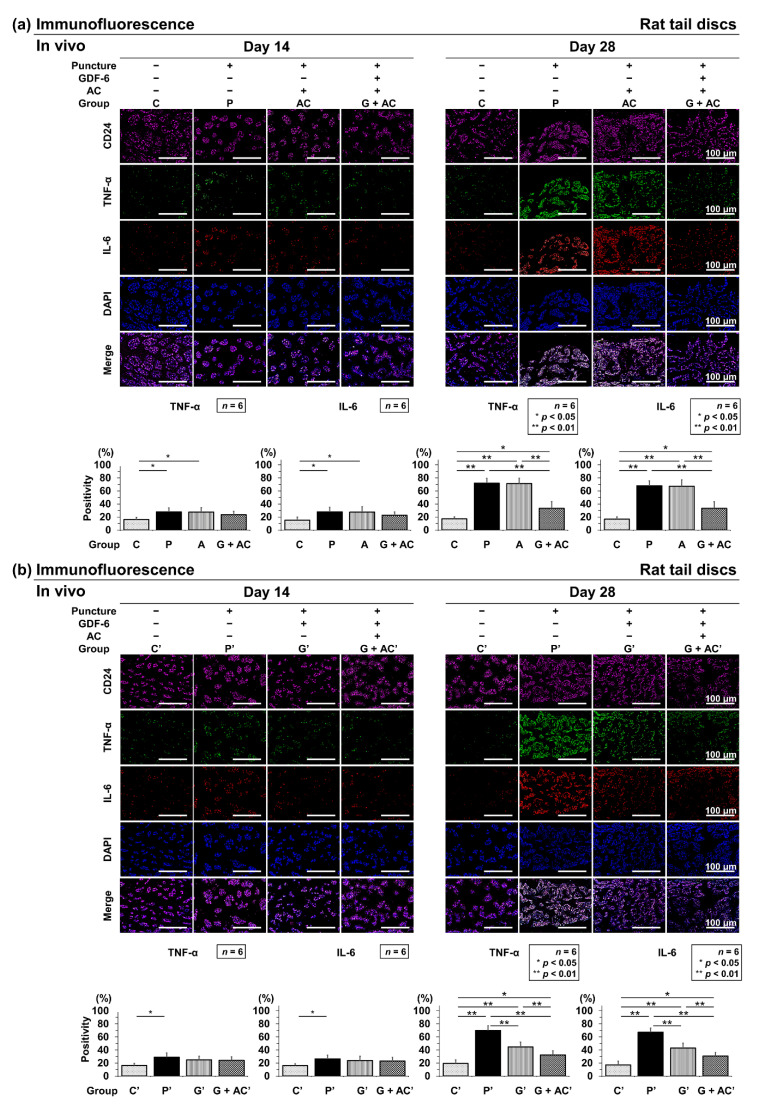
(**a**) Effects of co-administration of growth differentiation factor-6 (GDF-6) and atelocollagen (AC) on tumor necrosis factor-α (TNF-α) and interleukin (IL)-6 expression compared with AC alone in a rat tail puncture model. After establishment of the intervertebral disc (IVD) degeneration model, vehicle (phosphate-buffered saline [PBS]; 2 μL per disc, group P), AC (2-μL AC per disc, group AC), or GDF-6 (20 μg in 2-μL AC gel per disc, group G + AC) was randomly injected. Pro-inflammatory cytokines were assessed using immunofluorescence (TNF-α: green, IL-6: red, CD24: purple, 4′,6-diamidino-2-phenylindole [DAPI]: blue, and merged signals) of the IVDs at 14 and 28 days after injection in high-power fields (×400). Immunopositivity was calculated as a percentage relative to DAPI-positive cells. We designated the control without puncture (groups C and C′), and compared the four groups (C, P, A, G + AC) and (C′, P′, G′, G + AC′). Data are expressed as the mean ± standard deviation (*n* = 6). One-way analysis of variance (ANOVA) with the Tukey–Kramer post-hoc test was used. Significant differences are set as * *p* < 0.050 and ** *p* < 0.010. (**b**) Effect of co-administration of GDF-6 and AC on TNF-α and IL-6 expression compared with GDF-6 alone in a rat tail puncture model. After establishment of the IVD degeneration model, vehicle (PBS; 2 μL per disc, group P′), GDF-6 (20 μg in 2-μL PBS per disc, group G′), or GDF-6 (20 μg in 2-μL AC gel per disc, group G + AC′) was randomly injected. Pro-inflammatory cytokines were assessed using immunofluorescence (TNF-α: green, IL-6: red, CD24: purple, DAPI: blue, and merged signals) of the IVDs at 14 and 28 days after injection in high-power fields (×400). Immunopositivity was calculated as a percentage relative to DAPI-positive cells. We designated the control without puncture (groups C and C′), and compared the four groups (C, P, A, G + AC) and (C′, P′, G′, G + AC′). Data are the mean ± standard deviation (*n* = 6). One-way ANOVA with the Tukey–Kramer post-hoc test was used. Significant differences are set as * *p* < 0.050 and ** *p* < 0.010.

Immunopositivity was calculated as a percentage of DAPI-positive cells. In the control, an abundance of TNF-α-positive cells was observed at both time points (C: 14 days, 16.2 ± 3.4%; 28 days, 17.2 ± 2.9%) (Figure 8a) and C′: 14 days, 16.2 ± 2.9%; 28 days, 19.2 ± 5.5%) (Figure 8b). Then, IL-6-positive cells were also abundant at both time points (C: 14 days, 15.0 ± 4.8%; 28 days, 16.57 ± 3.4%) (Figure 8a) and C′: 14 days, 16.0 ± 3.0%; 28 days, 17.2 ± 5.4%) (Figure 8b).

However, 14 days after puncture, there was a significant increase in the number of TNF-α-positive cells in groups P and AC, although no significant difference was observed in group G + AC compared to group C (P: 28.0 ± 6.0%, AC: 27.1 ± 7.0%, G + AC: 23.5 ± 5.3%) (Figure 8a). Similarly, there was a predominant increase in the number of TNF-α-positive cells in group P′, although no significant increase was observed in groups G′ and G + AC′ compared to group C′ (P′: 29.0 ± 6.5%, G′: 24.9 ± 5.5%, G + AC′: 23.1 ± 5.4%) (Figure 8b). Then, IL-6-positive cells presented consistent results with TNF-α-positive cells (P: 27.9 ± 7.0%, AC: 27.9 ± 7.8%, G + AC: 22.6 ± 5.1%, P′: 26.3 ± 5.8%, G′: 24.0 ± 6.4%, and G + AC′: 23.1 ± 5.4%) (Figure 8a,b).Twenty-eight days after puncture, the increase in group G + AC compared to in groups P (TNF-α; *p* < 0.001, IL-6; *p* < 0.001) and AC (TNF-α; *p* < 0.001, IL-6; *p* < 0.001) (Figure 8a) and that in groups P′ (TNF-α; *p* < 0.001, IL-6; *p* < 0.001) and G′ (TNF-α; *p* = 0.038, IL-6; *p* = 0.022) compared to in group G + AC′ were significantly suppressed. Furthermore, the increase in group G′ was significantly suppressed compared to in group P′ (TNF-α; *p* < 0.001, IL-6; *p* < 0.001) (Figure 8b).

#### 3.3.5. Effects of Co-Administration of GDF-6 and AC on p-p38 Expression in a Rat Tail Puncture Model

**Figure 9:**
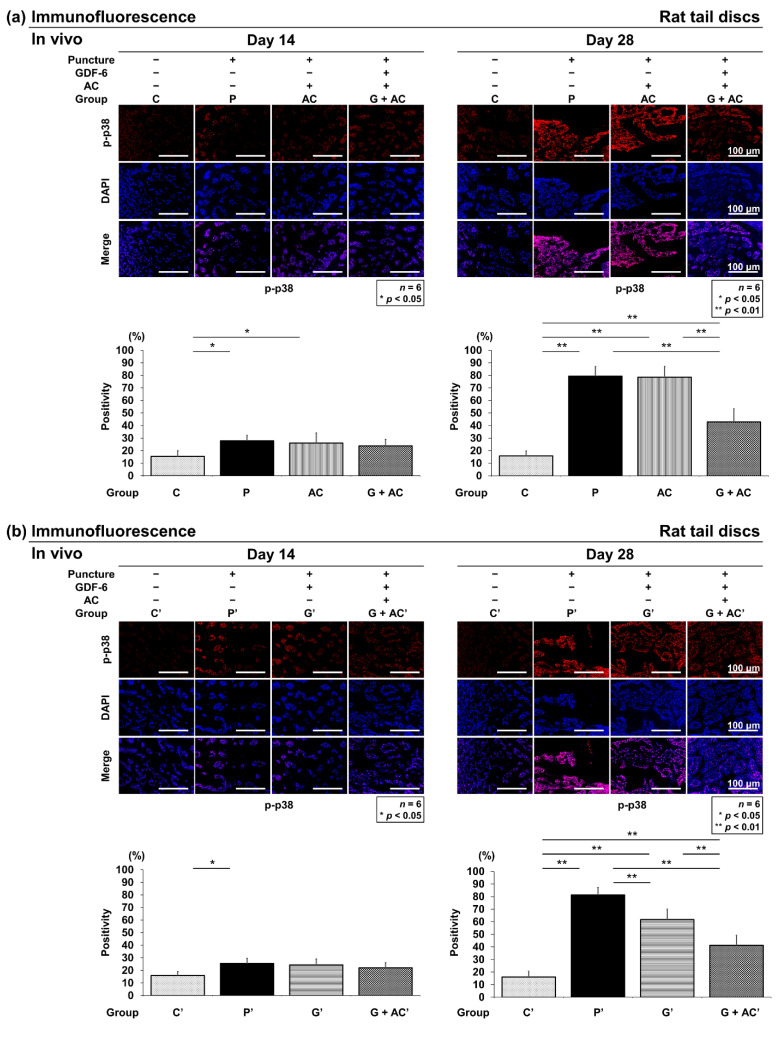
(**a**) Effects of co-administration of growth differentiation factor-6 (GDF-6) and atelocollagen (AC) on p-p38 expression compared with AC alone in a rat tail puncture model. After establishment of the intervertebral disc (IVD) degeneration model, vehicle (phosphate-buffered saline [PBS]; 2 μL per disc, group P), AC (2-μL AC per disc, group AC), or GDF-6 (20 μg in 2-μL AC gel per disc, group G + AC) was randomly injected. Pro-inflammatory cytokines were assessed using immunofluorescence (p-p38: red, CD24: purple, 4′,6-diamidino-2-phenylindole [DAPI]: blue, and merged signals) of the IVDs at 14 and 28 days after injection in high-power fields (×400). Immunopositivity was calculated as a percentage relative to DAPI-positive cells. We designated the control without puncture (groups C and C′), and compared the four groups (C, P, A, G + AC) and (C′, P′, G′, G + AC′). Data are expressed as the mean ± standard deviation (*n* = 6). One-way analysis of variance (ANOVA) with the Tukey–Kramer post-hoc test was used. Significant differences are set as * *p* < 0.050 and ** *p* < 0.010. (**b**) Effect of co-administration of GDF-6 and AC on p-p38 expression compared with GDF-6 alone in a rat tail puncture model. After establishment of the IVD degeneration model, vehicle (PBS; 2 μL per disc, group P′), GDF-6 (20 μg in 2-μL PBS per disc, group G′), or GDF-6 (20 μg in 2-μL AC gel per disc, group G + AC′) was randomly injected. Pro-inflammatory cytokines were assessed using immunofluorescence p-p38: red, CD24: purple, DAPI: blue, and merged signals) of the IVDs at 14 and 28 days after injection in high-power fields (×400). Immunopositivity was calculated as a percentage relative to DAPI-positive cells. We designated the control without puncture (groups C and C′), and compared the four groups (C, P, A, G + AC) and (C′, P′, G′, G + AC′). Data are the mean ± standard deviation (*n* = 6). One-way ANOVA with the Tukey–Kramer post-hoc test was used. Significant differences are set as * *p* < 0.050 and ** *p* < 0.010.

Immunopositivity was calculated as a percentage of DAPI-positive cells. In the control, an abundance of p-p38-positive cells was observed at both time points (C: 14 days, 15.5 ± 4.5%; 28 days, 15.8 ± 3.0%) (Figure 9a) and C′: 14 days, 15.9 ± 3.0%; 28 days, 16.1 ± 4.5%) (Figure 9b). However, 14 days after puncture, there was a significant increase in the number of p-p38-positive cells in groups P and AC, although no significant difference was observed in group G + AC compared to group C (P: 28.0 ± 4.2%, AC: 26.1 ± 8.0%, G + AC: 23.9 ± 5.1%) (Figure 9a). Similarly, there was a predominant increase in the number of p-p38-positive cells in group P′, although no significant increase was observed in groups G′ and G + AC′ compared to group C′ (P′: 26.2 ± 6.3%, G′: 23.1 ± 5.5%, G + AC′: 22.1 ± 4.1%) (Figure 9b). Twenty-eight days after puncture, the increase in group G + AC compared to that in groups P (*p* < 0.001) and AC (*p* < 0.001) (Figure 9a) and that in groups P′ (*p* < 0.001) and G′ (*p* < 0.001) compared to that in group G + AC′ were significantly suppressed. Furthermore, the increase in group G′ was significantly suppressed compared to in group P′ (*p* = 0.001) (Figure 9b).

## 4. Discussion

The BMP family, including GDF-6, influences the IVD homeostasis between anabolic factors and pro-inflammatory cytokines. Disrupting this balance leads to the progression of IVD degeneration. In this study, we examined the effects of GDF-6 according to age and IVD degeneration on IVD NP cells and observed the decreased expression of GDF-6 in IVD with aging and severity of degeneration. Although no difference in the expression of BMPRII (GDF-6 receptor) has been reported in the IVD owing to the degeneration [22], our results exhibited decreased levels of BMPs and BMPRII. Okuda et al. reported that the metabolic/anabolic responsiveness of IVD cells to growth factors decreases with age, which is associated with decreased growth factor production and receptor synthesis in IVD cells [40]. Thus, we compared two groups of patients, those with Pfirrmann grades II or III (moderate degeneration group) and with Pfirrmann grade IV (severe degeneration group), and identified the different responses to GDF-6 in Pfirrmann grade-IV patients from Pfirrmann grade-II or -III patients. While there have been no reports using TASCL in IVD cell culture, we used TASCL for 3D culture in the current study [41]. The TASCL has a two-layered structure with a lattice-shaped device and microporous membrane that facilitates the production of many uniform cell clusters using simple cell suspensions. This was considered more advantageous than the 3D culturing system using alginate beads, which involves a complicated procedure to obtain uniform cell clusters.

Treatment of GDF-6 for moderately degenerated IVDs increased the expression of notochordal CD24 and anabolic factors, such as aggrecan and type II collagen, which is consistent with a prior study [42]. In addition, we previously reported the decreased levels of IL-6 and TNF-α by GDF-6 in rabbit IVD studies in vivo [21]. However, no prior studies have compared the effects of GDF-6 on IVD cells under IL-1β-induced pro-inflammatory conditions using a 3D in vitro culturing system. In the current study, we showed in real-time RT–PCR that GDF-6 reduced the levels of TNF-α and IL-6 in IVD NP cells under pro-inflammatory stimulation. To corroborate these results, we focused on the MAPK pathway enhanced by GDF-6 administration. Our results showed that GDF-6 inhibited the IL-1β stimulated p38 phosphorylation. Hence, although the underlying mechanism associated with the efficacy of GDF-6 under pro-inflammatory stimulation requires further investigation, GDF-6 may be a potent therapeutic target for the management of inflammation and pain during the early stages of IVD degeneration.

For the in vivo experiments, we selected the rat caudal annular puncture model, which is a simple model for creating degenerated IVDs. Moreover, we employed the 30 s fixation technique with a 360° rotation by advancing a 20-G needle to 5-mm depth, as described previously [37]. The DHI after puncture with a 20-G needle was slightly lower than that reported with this method at two and four weeks [37]. However, a similar decrease was obtained with a less invasive puncture model using a 21-G needle with 180° rotation and 5-s fixation [43]. Therefore, the puncture technique was appropriate and effective for the purpose of this study.

We hypothesized that locally administered drugs or growth factors via injection or surgery to IVDs, which can be nourished only by diffusion, would not be effective since they could easily flow out of the intradiscal space. The AC has been investigated as an injectable scaffold [26,44]; AC solution becomes liquid at nearly 4 °C and gel at 37 °C, which may be a significant advantage in keeping the drug or growth factor localized. Drug delivery systems (DDS) using collagen can overcome the shortcomings of water-soluble polymers in terms of instability. Collagen preparations have been reported to achieve the sustained release of various proteins and could enhance the effects of drugs locally [27]. Type II collagen-based AC gel has a similar composition to IVD matrix; therefore, we selected it as a solvent owing to its low immunogenicity and high safety [26]. In vitro studies on culturing chondrocytes in AC gel have resulted in greater matrix synthesis compared to monolayer cell culture [26]. Porous hydroxyapatite/AC composites for treating bone defects are also often used clinically. Additional matrix is synthesized more abundantly when chondrocytes are cultured with AC than when cultured in a monolayer [45]. In this study, we focused on whether this property of AC could enhance the effect of GDF-6 by acting as a scaffold for NP cells.

AC could be injected without clogging or coagulation using a microsyringe. GDF-6 was soluble in AC, and the concentration was equalized by repetitive pipetting. The drug dose was set to 20 μg/disc based on the body weight ratio of rabbits and rats.

In vivo experiments in sheep and rabbits have identified that GDF-6 not only affects ECM synthesis but also suppresses the levels of pro-inflammatory cytokines, and is effective for maintaining IVD structure and treating discogenic pain [21]. In addition, GDF6 can improve the structure of the IVD, inhibit the expression of inflammatory and pain-related factors, and improve pain behavior in rats [46]. However, there have been no reports on GDF-6 administration combined with AC as a DDS. In our study, GDF-6 with AC had a more positive prolonged effect on IVD disruption by downregulating the inflammatory response and upregulating ECM production than GDF-6 alone. Our findings suggest that GDF-6 administered with AC can provide an IVD-protective effect with a relatively low dose of GDF-6 [46]. The use of AC as a DDS prolonged the effect of GDF-6 (compared with PBS) and possibly reduced the mechanical stress between vertebrae.

Although the protective effects of GDF-6 on IVDs have been reported [20,21,22,23], there have been no reports evaluating the effects of GDF-6 under inflammatory conditions in vitro, simulating IVD degeneration in vivo, and actually examining these effects in an in vivo degeneration model. Thus, this study has a high novelty with consistent results in vitro and in vivo.

This study has several limitations. First, we did not determine why GDF-6 exerted its anti-inflammatory effect only under pro-inflammatory conditions. Second, this study adopted a single concentration/viscosity of AC with no other scaffold controls. We did not investigate how GDF-6 release was sustained. In the future, it will be necessary to clarify the optimal conditions under which GDF-6 exerts its effects and to adjust the optimal scaffold.

## 5. Conclusions

The co-administration of GDF-6 and AC could decelerate degenerative IVD disease, which could serve as a new treatment strategy for degenerative IVD disease.

## Figures and Tables

**Table 1:** Primer sequences used in the study.

Gene Name	Forward Primer Sequence (5′–3′)	Reverse Primer Sequence (5′–3′)
*ACAN*	AAGAATCAAGTGGAGCCGTGTGTC	TGAGACCTTGTCCTGATAGGCACT
*COL2A1*	AAGGTGCTTCTGGTCCTGCTG	GGGATTCCATTAGCACCATCTTTG
*TNFA*	GTGACAAGCCTGTAGCCCATGTT	TTATCTCTCAGCTCCACGCCATT
*IL-6*	AAGCCAGAGCTGTGCAGATGAGTA	TGTCCTGCAGCCACTGGTTC
*MMP-3*	CAAGGAGGCAGGCAAGACAGC	GCCACGCACAGCAACAGTAGG
*ADAMTS-4*	GGATTACAGGTGTGAGCCACCA	GGATGCAACCACATCTGTCTGA
*GAPDH*	GAGGCCGGTGCTGAGTAT	GCGGAGATGATGACCCTTTTGG

*ACAN* = aggrecan; *ADAMTS* = a disintegrin and metalloproteinase with thrombospondin motifs; *COL2A1* = collagen type II alpha 1 chain; *GAPDH* = glyceraldehyde 3 phosphate dehydrogenase; IL = interleukin; *MMP* = matrix metalloproteinase; *TNFA* = tumor necrosis factor-α.
